# Histopathological characteristics of PRRS and expression profiles of viral receptors in the piglet immune system

**DOI:** 10.3389/fvets.2024.1428273

**Published:** 2024-11-06

**Authors:** Hong Chen, Na Chen, Hongbo Chen, Zefang Zhao, Jiayao Yang, Jianbo Sun, Hanmei Li, Rihua Cong, Hailong Liu, Tengfei Liu, Shulin Chen

**Affiliations:** ^1^Department of Basic Veterinary Medicine, College of Veterinary Medicine, Northwest A&F University, Yangling, China; ^2^Institute of Animal Science and Veterinary Medicine, Hainan Academy of Agricultural Sciences, Haikou, China

**Keywords:** PRRSV, immune organs, viral receptors, histopathology, piglets

## Abstract

Porcine reproductive and respiratory syndrome (PRRS) is a highly contagious viral disease that causes significant economic losses to the swine industry worldwide. PRRS virus (PRRSV) infection is a receptor-mediated endocytosis and replication process. The purpose of this study was to determine the localization and expression of four important PRRSV receptors in immunological organs of piglets. After piglets were infected with PRRSV, Hematoxylin and Eosin staining, immunofluorescence, and Western blot were used to perform histopathological examination and receptors distribution analysis. The results showed that PRRSV caused severe damage to the piglets’ immune organs, including atrophy of the thymus and swelling of lymph node. Histopathological lesions were mainly observed in the lung and lymph node and were characterized by interstitial pneumonia, collapsed follicles, exhaustion of germinal centers, and extensive hemorrhage. Immunofluorescence staining and Western blot results showed that the receptors of CD163 and NMHCII-A were mainly distributed in the thymus, hilar lymph nodes, and mesenteric lymph nodes. However, Sn and vimentin receptors were expressed at low levels in the immune organs of piglets. The distribution of the four receptors in the immune organs was more concentrated in the cortex but was more scattered in the medulla. Compared to the control group, the relative expression of the four receptors increased significantly in most immune organs after viral infection. In conclusion, our study examined the distribution and expression of four PRRSV receptors in immunological organs. We observed a significant increase in the expression of Sn, CD163, and vimentin following viral infection. These findings may provide potential targets for future antiviral reagent design or vaccine development.

## 1 Introduction

Porcine reproductive and respiratory syndrome (PRRS), also known as blue-eared pig disease in China, has been an epidemic in the United States for more than 30 years and in China for 20 years (1). PRRS leads to reproductive complications, including embryonic mortality, late-stage abortion, preterm birth, increased incidence of stillborn and mummified fetuses, as well as the birth of weak piglets (2). The etiological agent of this disease is PRRSV, an enveloped RNA virus that belongs to the genus and family of *Arterivirus* (3). The PRRSV genome is approximately15 kb in length and has at least 10 open reading frames (ORFs) encoding 16 nonstructural and 8 structural proteins (4). Based on its antigenicity, PRRSV is divided into two species, PRRSV-1 (*Betaarterivirus suid 1*, formerly known as European genotype 1) and PRRSV-2 (*Betaarterivirus suid 1*, formerly known as North American genotype 2), of which only 50–70% are present a common nucleotide sequence identity (5). In China, PRRSV-2 is the most prevalent genotype in pig farms and is further divided into lineage 1 (represented by NADC30 and NADC 34), lineage 3 (represented by QYYZ), lineage 5 (represented by VR-2332), and lineage 8 (represented by JXA1) based on their ORF5 sequence (6). Studies have shown that recombination strains from different lineages are becoming the predominant strains in China, especially NADC30-like strains (7, 8). Importantly, PRRSV is currently spreading around the world. Recent studies have shown a prevalence of 16.7% in Campaina (Italy) and 58% in Costa Rica (9, 10).

Previous research has shown that PRRSV remains in the blood, spleen, lymph nodes and tonsils of infected pigs (11). PRRSV infection results in chronic viral infection due to an inefficient cellular immune response and exhibits restricted cell tropism, with alveolar macrophages being the primary target cells (12). The intensity and susceptibility to PRRSV infection varies with age (13, 14). Younger piglet macrophages were more susceptible to PRRSV infection, although there was no change in the expression of surface receptors in pigs of different ages (15). PRRSV infection is actually a host cell receptor-mediated endocytosis and replication process. The presence or absence of the cellular receptors determines whether the virus can penetrate the host cells (16). To date, at least six cellular receptors have been identified as viral entry mediators for PRRSV, including sialoadhesin (Sn, CD169), vimentin, nonmuscular myosin heavey chain II subtype A (NMHC-II-A), heparin sulfate, dendritic cell-specific intercellular adhesion molecule-3-grabbing non-integrin (CD209), and CD163 (cysteine-rich scavenger receptor) (17, 18). Each receptor has its own characteristics in term of distribution in different cells, function at different stages of viral infection, and interaction patterns with viral proteins or genes (19). Brianna Salgado and colleagues recently discovered that genomic editing of the host receptor CD163 can be used as a strategy to create genetically engineered pigs that are completely resistant to PRRSV infection (20). Therefore, systematic studies on the distribution and localization properties of PRRSV receptors have become a new strategy for the prevention of PRRSV infection.

The present investigation determined the distribution of four PRRSV receptors (Sn, CD163, vimentin, and NMHCII-A) in the immunological organs of piglets after PRRSV infection. Our results showed that four receptors were widely distributed in the thymus, tonsil, inguinal lymph, spleen, hilar lymph nodes, mesenteric lymph, and mandibular lymph. The expression of the Sn, CD163, and vimentin increased significantly after PRRSV infection compared to the control group. Our morphological results provide evidence of interactions between viruses and immune organs and are valuable for identifying the mechanisms of virus infection pathways.

## 2 Materials and methods

### 2.1 Animals and infection

Ten crossbred piglets (30 days old, female: male = 5:5) were used in the present study. The piglets were fed an antibiotic-free diet and had unrestricted access to water. After regular serological testing, all piglets were confirmed to be free of PRRSV, PPV, PCV, and swine influenza virus (SIV) before their use in this study. Piglets were randomly assigned to either the control group or the PRRSV infection group (each group: 5 piglets). All piglets were first injected into the neck muscles with an immunologic adjuvant (0.11 mg/kg) at 0 DPI and 14 DPI (d post-infection). After 28 DPI, piglets in the infection group were administered cell supernatant of Marc-145 cells containing PRRSV-2 JXA1 (lineage 8, 10^−5^ TCID_50_/0.1 mL) on the 0^th^ injection day post-challenge (DPC) by intranasal administration (0.3 mL per piglets). The same volume of PRRSV-free cell supernatant was administrated to the control group. Every day between 0 and 28 DPC, all piglets underwent daily clinical examinations to monitor their overall behavior, appetite, rectal temperature, respiratory rate, and any signs of respiratory disease or diarrhea. Blood samples were collected at 0, 7, 10, 14, 21, and 28 DPC to determine GP5 and protein N antibody levels. All piglets were slaughtered at 28 DPC for gross and histopathological examination. For further analysis, immunological samples were immediately collected from the tonsils, thymus, inguinal lymph nodes, mesenteric lymph nodes, hilar lymph nodes, spleen, and mandibular lymph nodes.

### 2.2 Indirect ELISA analysis

PRRSV is a small, enveloped virus with six structural proteins (GP2, GP3, GP4, GP5, M, and N). The GP5 and N proteins are essential targets for serological detection using ELISA. There are currently two widely used commercial kits for diagnosing PRRSV antibodies: IDEXX HerdChek PRRS Ab X3 (IDEXX Laboratories, Westbrook, Maine, United States) and LSI Porcine PRRS/AS-serum (LSI-ELISA from LSI company of France). There are significant differences between the two kits. The IDEXX-ELISA uses plates coated with the virus’s nucleocapsid protein (N protein), while the LSI-ELISA kit uses the viral glycoprotein (GP5) (21). The LSI-ELISA method considers samples positive if the relative index percent (IRPC) is ≥20 and negative if it is <20. The IRPC is calculated by (Sample OD_450_-negative control OD_450_)/ (Positive OD_450_-negative-control OD_450_) × 100. IDEXX-ELISA method considers samples positive if the S/P ratio is ≥0.4 and negative if it is <0.4, where S/P = (Sample OD_650_-negative control OD_650_)/ (Positive OD_650_-negative-control OD_650_). Serum samples from piglets were analyzed for anti-PRRVS antibodies using the two commercial indirect ELISA kits mentioned above. The manufacturer’s instructions were followed to ensure rapid and accurate diagnosis of PRRSV in the study.

### 2.3 Histopathological examination

At the end of the experiment, tissue samples from various immune organs, including thymus, tonsil, inguinal lymph nodes, spleen, and hilar lymph nodes, were quickly collected and fixed in 10% neutral buffered formalin (v/v) overnight at room temperature before being embedded in paraffin wax. The wax blocks containing the samples were prepared and then cut into 5 μm thick sections. These sections were subjected to staining with the hematoxylin and eosin (H&E) technique and then examined under a light microscope equipped with an Olympus DP73 (Tokyo, Japan).

### 2.4 Immunohistochemistry

The tissue sections of the immune organ were deparaffinized in xylene and then rehydrated with a series of alcohol solutions. To neutralize the endogenous peroxidase activity, the sample sections were immersed in a 3% hydrogen peroxide solution for 10 min. After three washes with PBS, antigen retrieval was carried out by boiling the sample sections in sodium citrate buffer for 10 min. To prevent non-specific binding, sample sections were then treated with 5% bovine serum albumin (BSA) for 30 min before being incubated overnight at 4°C with the primary antibody (Anti-PRRS virus Nucleocapsid antibody, abcam, ab308202). After washing with PBS, biotinylated secondary antibody was applied to the sample sections and incubated for 30 min at room temperature. Sample sections were rinsed three times with PBS and positive reactions were visualized using a DAB kit.

### 2.5 Immunofluorescence

After incubation with 5% bovine serum albumin (BSA) for 30 min to prevent non-specific binding, the sample sections were incubated overnight at 4°C with the primary antibody of Anti CD163 (Affinity, DF8235), Sn (Affinity, DF13669), NMHCII-A (Abcam, ab138498) and vimentin (Affinity, BF8006). After three washes with PBS, Alexa Fluor 488-labeled IgG was added and incubated for 1 h at 37°C. The sample sections were subjected to three cycles of cleaning before being stained with DAPI. Finally, confocal laser scanning microscopy (Leica TCS SP8) was used to observe the sections.

### 2.6 Western blotting

The immunological samples were collected and immediately placed on ice, followed by a cold PBS wash. The samples were then homogenized in a cell lysis buffer (RIPA buffer, Beyotime, P0013) containing protease and phosphatase inhibitors. After centrifugation at 12,000 × g for 15 min, the supernatants were obtained. Protein concentration was determined using a BCA protein assay kit (Solarbio, PC0020) and equal amounts of protein samples were separated via SDS-PAGE on a 12% gel. After electrophoretic transfer of proteins to a PVDF membrane (Millipore), the membrane was blocked for 1 h with 5% (w/v) skim milk powder in Tris-buffered saline containing 0.05% Tween-20 at room temperature. Next, primary antibodies (CD163, Sn, NMHCII-A, and vimentin) were incubated on the membranes overnight at 4°C. After the TBST wash steps, appropriate secondary antibodies were applied to the membranes for 1 h at room temperature. The immunoreactive bands were examined using an enhanced chemiluminescence (ECL) detection system (Vazyme Biotech, China) and Quantity One software (Bio-Rad Laboratories) to quantify the immunoreactive bands.

### 2.7 Statistical analysis

Data were presented as means ± SEM. Statistical significance of mean differences between groups was determined using ANOVA with a multiple comparison test performed in GraphPad Prism 7.0. All quantification data were repeated three times and the results were considered statistically significant when the *p*-value was less than 0.05 (*) or 0.01 (**).

## 3 Results

### 3.1 Gross pathological examination and humoral immune response

**Figure 1:**
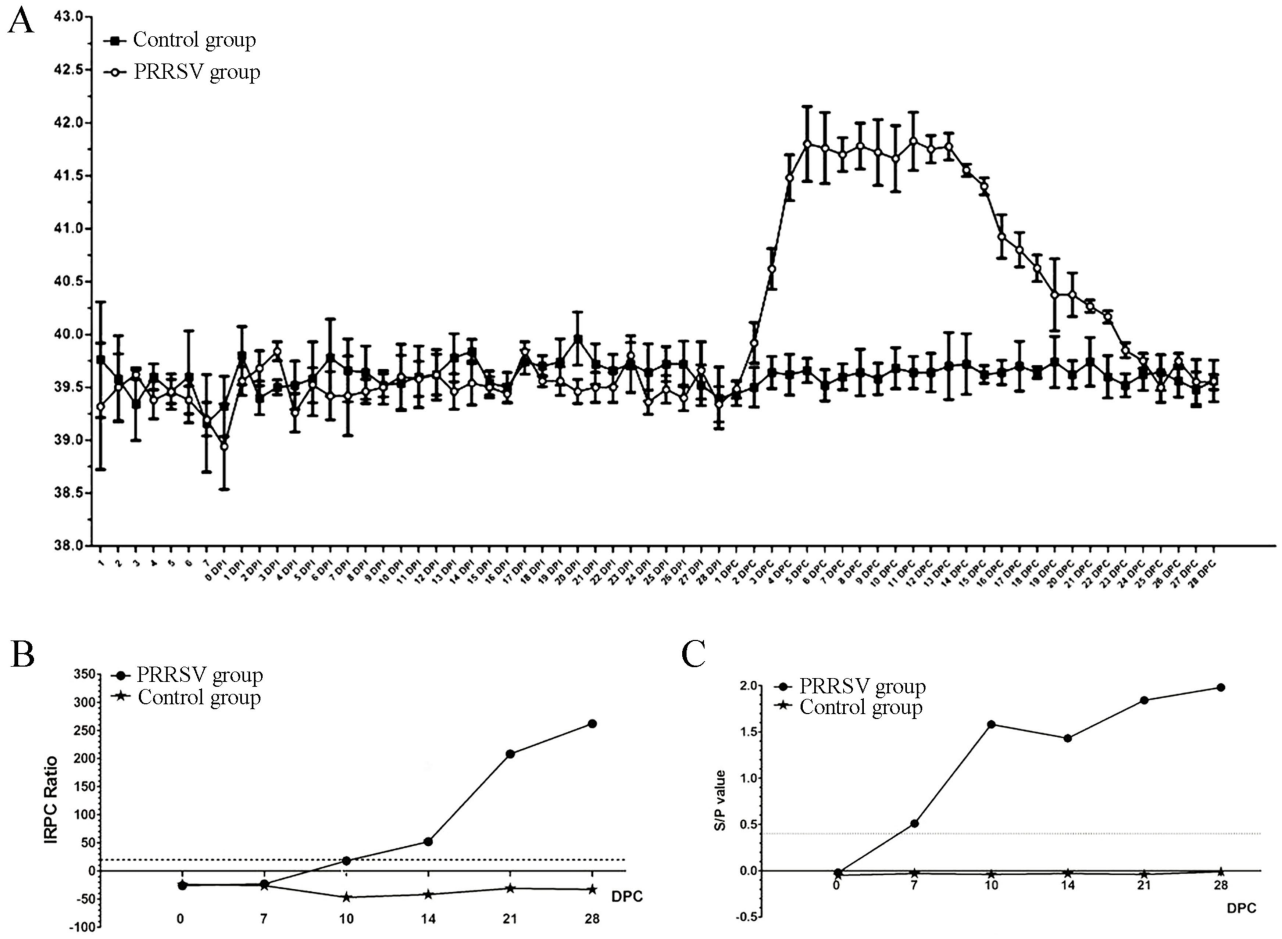
Effect of PRRSV infection on piglets. **(A)** The rectal temperature of piglets in control group and PRRSV group. **(B)** The blood level of the PRRSV-specific antibody for GP5 was detected by LSI-ELISA kit. **(C)** The blood level of the PRRSV-specific antibody for protein N was detected by IDEXX-ELISA kit.

The piglets in the control group did not show any typical clinical symptoms during the experiment. By the 2nd DPC, there were no changes in the clinical signs of respiratory disease between the control and infected piglets. Almost all infected piglets in the 3rd DPCs exhibited coughing, sneezing, loss of appetite and diarrhea. These clinical symptoms persisted until the animal experiment was stopped. The rectal temperature of all control piglets remained constant at 39.5°C throughout the experiment (Figure 1A). However, the rectal temperature of the infected piglets increased to 41°C at the 4th DPC and remained above 41°C for 12 days (Figure 1A). Next, we used an indirect ELISA approach to measure blood levels of the PRRSV-specific antibodies for GP5 and protein N. The results showed that the positive rate increased significantly at the 10th DPC as determined by the LSI-ELISA kit (Figure 1B). At the 7th DPC, the S/*p* value increased more significantly with the addition of the IDEXX-ELISA kits (Figure 1C). The control piglets remained seronegative until the end of the study.

**Figure 2:**
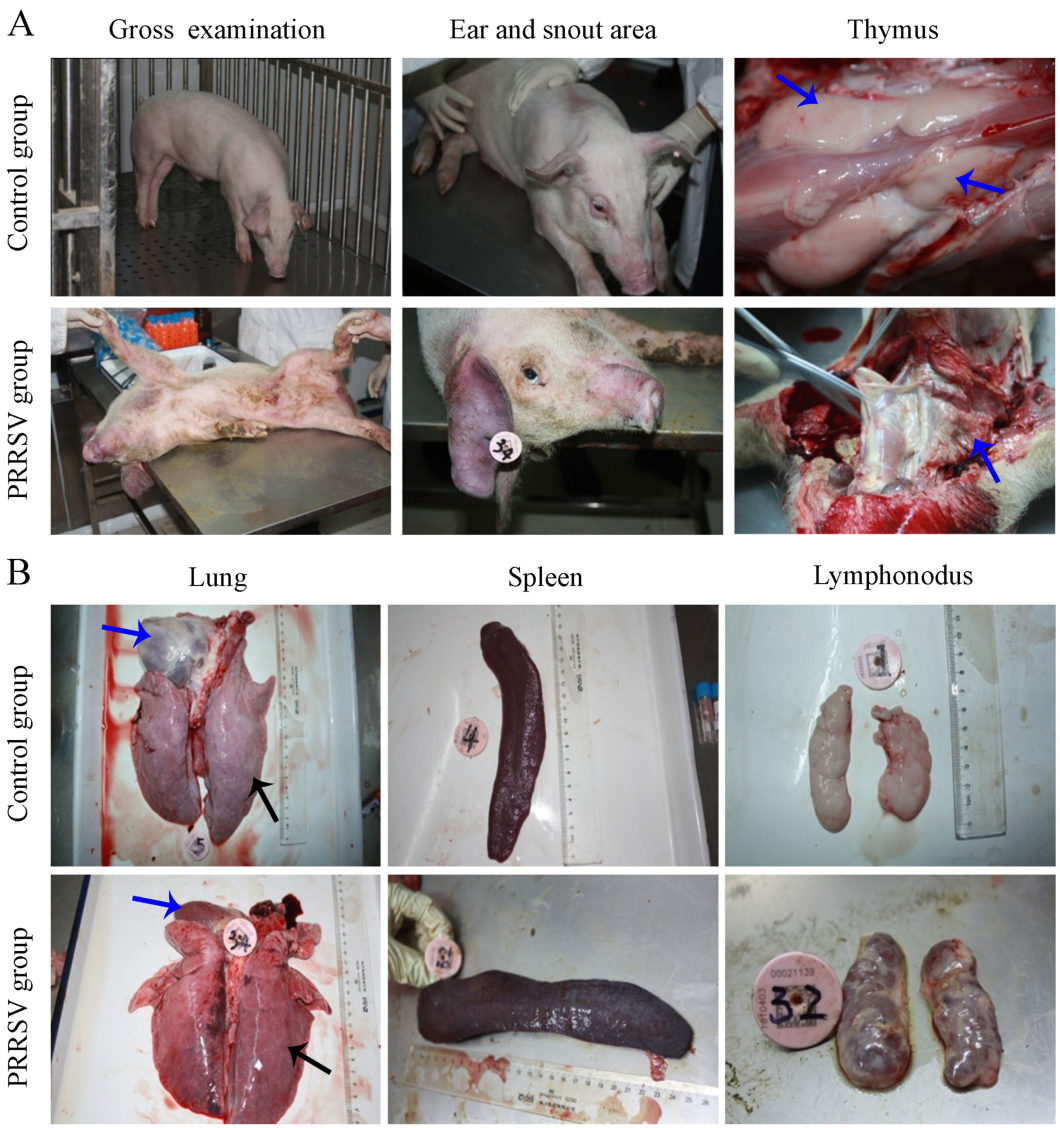
Gross pathological examination of infected piglets. **(A)** The cyanosis in the skin of the ear and snout area was observed in infected piglets. **(B)** Pathological lesions were observed in visceral organs and immune organs.

In addition, the skin of the hindquarter and abdomen was erythematous, with obvious cyanosis in the skin of the ear and snout area (Figure 2A). Pathological lesions were observed, mainly located in the lungs and lymph nodes. The thymus gland of the infected piglets was atrophied and covered with hemorrhagic spots (Figure 2A). Interstitial pneumonia and systemic expansion of the inguinal, mesenteric, hilar, and mandibular lymph nodes were the most significant pathological changes. Compared with those of the control group, the lungs of infected piglets were diffusely reddened, non-collapsing, hard and rubbery (Figure 2B). White nodules were found in the apical lobe and diaphragmaticus lobe of the infected lungs. The heart chambers were blocked and there were inflammatory fluids in the pericardium. The spleen of the piglets in the infection group was enlarged and covered with red nodules (Figure 2B). Compared to control piglets, the infected piglets had firmer, hemorrhagic, and larger hilar, mesenteric and inguinal lymph nodes (Figure 2B).

### 3.2 Histopathological examination and virus detection in tissues

**Figure 3:**
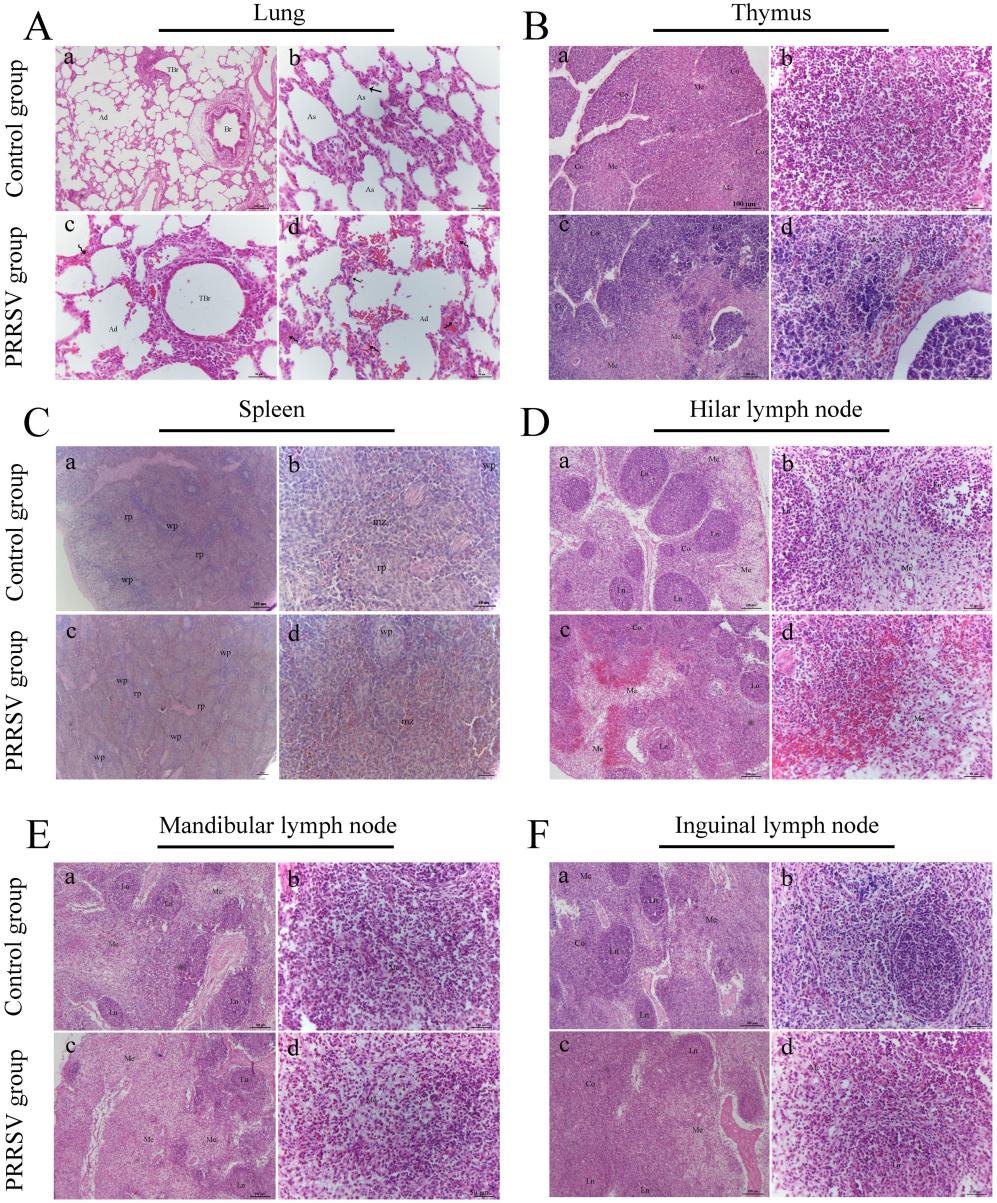
Histopathological observation in infected piglets. **(A)** The histopathological observation in the lungs; Br: bronchiole; TBr: terminal bronchiole; Ad: alveolar duct; As: alveolar sac; Curved arrow: alveolar septum; Black arrow: type II alveolar cell; Scale bars a = 100 μm; b, c, and d = 50 μm. **(B)** The histopathological observation in the thymus; Co: cortex; Me: medulla; Scale bars a, c = 100 μm; b, d = 50 μm. **(C)** The histopathological observation in the spleen; wp: white pulp; rp: red pulp; mz: marginal zone; Scale bars a, c = 200 μm; b, d = 50 μm. **(D)** The histopathological observation in the hilar lymph node; Co: cortex; Me: medulla; Ln: lymphatic nodule; Scale bars a, c = 100 μm; b, d = 50 μm. **(E)** The histopathological observation in the mandibular lymph node; Me: medulla; Ln: lymphatic nodule; Scale bars a, c = 100 μm; b, d = 50 μm. **(F)** The histopathological observation in the inguinal lymph node; Co: cortex; Me: medulla; Ln: lymphatic nodule; Scale bars a, c = 100 μm; b, d = 50 μm.

**Figure 4:**
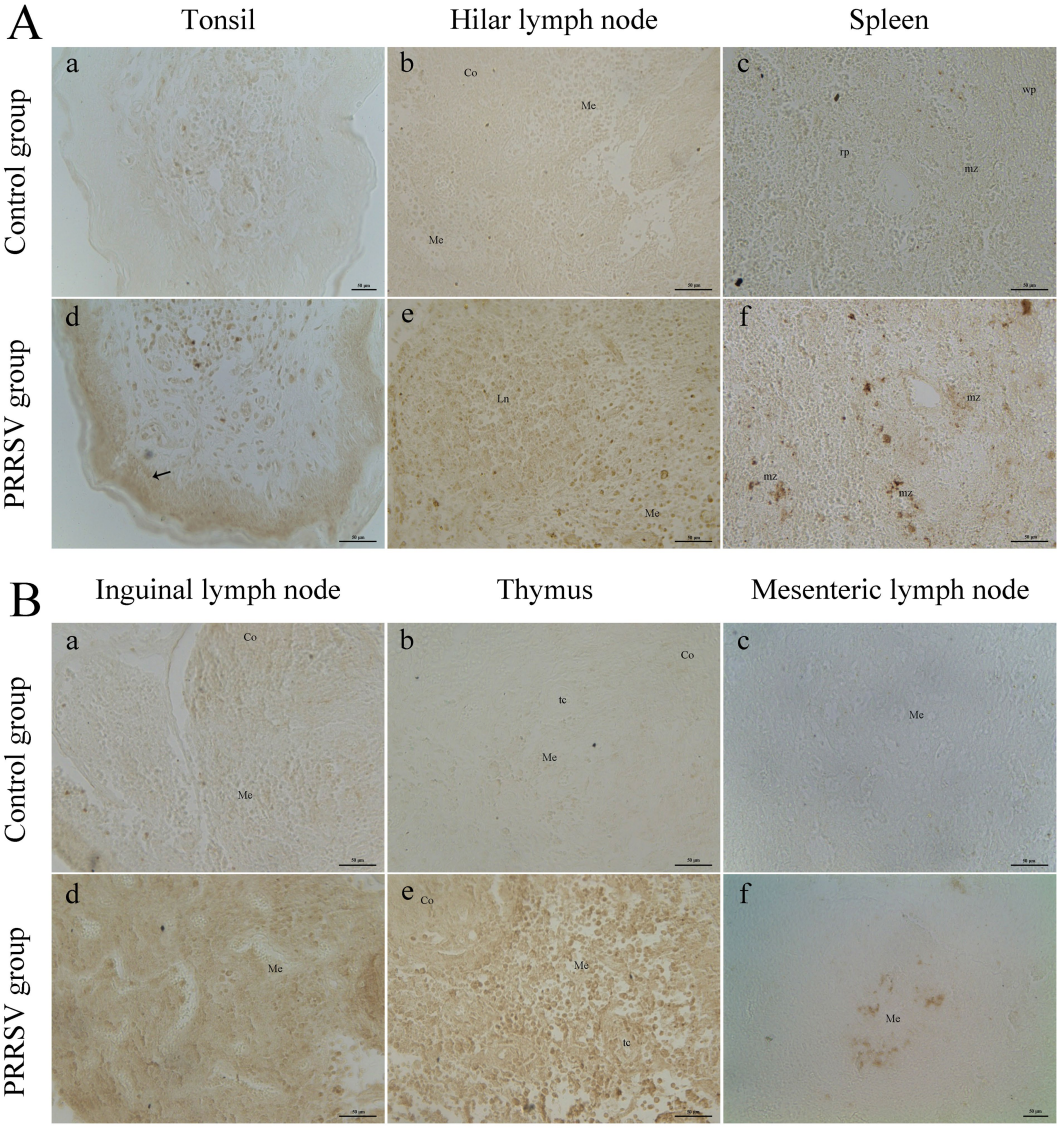
The distribution of PRRSV in immune organs was analyzed by immunohistochemistry. **(A)** The immune organs of tonsil, hilar lymph node, and spleen were analyzed. Co: cortex; Me: medulla; Ln: lymphatic nodule; wp: white pulp; rp: red pulp; mz: marginal zone; Black arrow: lymphatic tissue in tonsil; Scale bars = 50 μm. **(B)** The immune organs of inguinal lymph node, thymus, and mesenteric lymph node were analyzed. Co: cortex; Me: medulla; tc: thymic corpuscle; Scale bars = 50 μm.

Compared to those of the control group, the lungs of infected piglets had different histological lesions. Diffuse interstitial pneumonia was characterized by significant alveolar duct hemorrhage and macrophage thickening of the alveolar septa, and occasionally by type II pneumocytes hypertrophy (Figure 3A). The thymus of infected piglets was characterized by a reduced number of lymphocytes. The lymphocytes in the cortical area were aggregated into small punctums and the space between cells was expanded, with some hemorrhagic spots in the medullary area of the thymus (Figure 3B). Histopathological lesions in the spleen and hilar, mandibular, and inguinal lymph nodes included collapsed follicles, exhaustion of germinal centers, and extensive hemorrhage (Figures 3C–F). We next used immunohistochemistry to detect the distribution of PRRSV in immunological organs (Figure 4). A stronger positive reaction was observed in the tonsil, hilar lymph nodes, mesenteric lymph nodes, medullary region of the thymus, and marginal zone of the spleen (Figures 4A,B). A relatively weak positive reaction was observed in the inguinal lymph nodes (Figure 4B).

### 3.3 Distribution and expression of PRRSV receptors in immune organs

**Figure 5:**
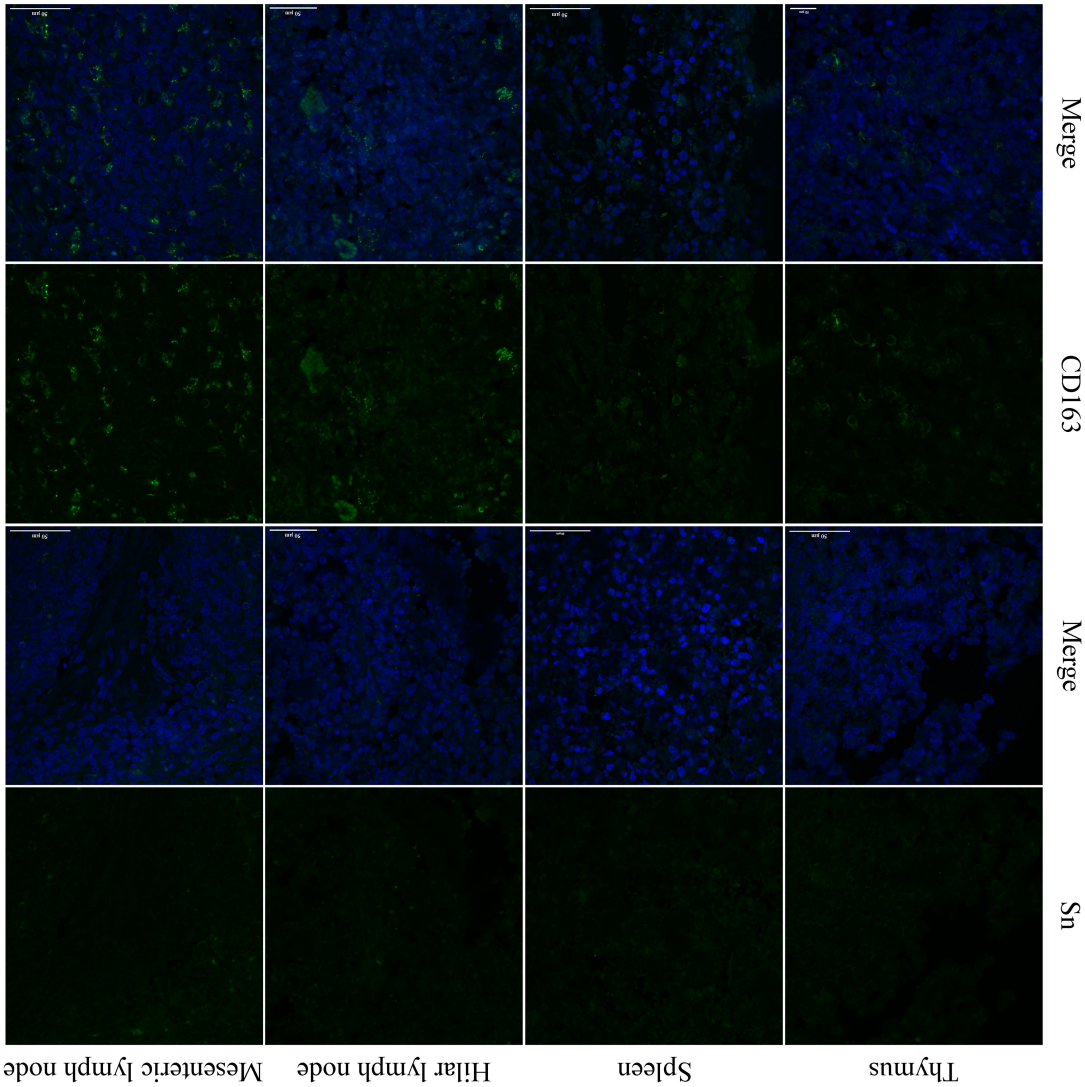
The distribution of PRRSV receptors in immune organs was analyzed by immunofluorescent staining. The receptors of Sn and CD163 were analyzed in thymus, spleen, hilar lymph node, and mesenteric lymph node, respectively. Scale bars = 50 μm.

**Figure 6:**
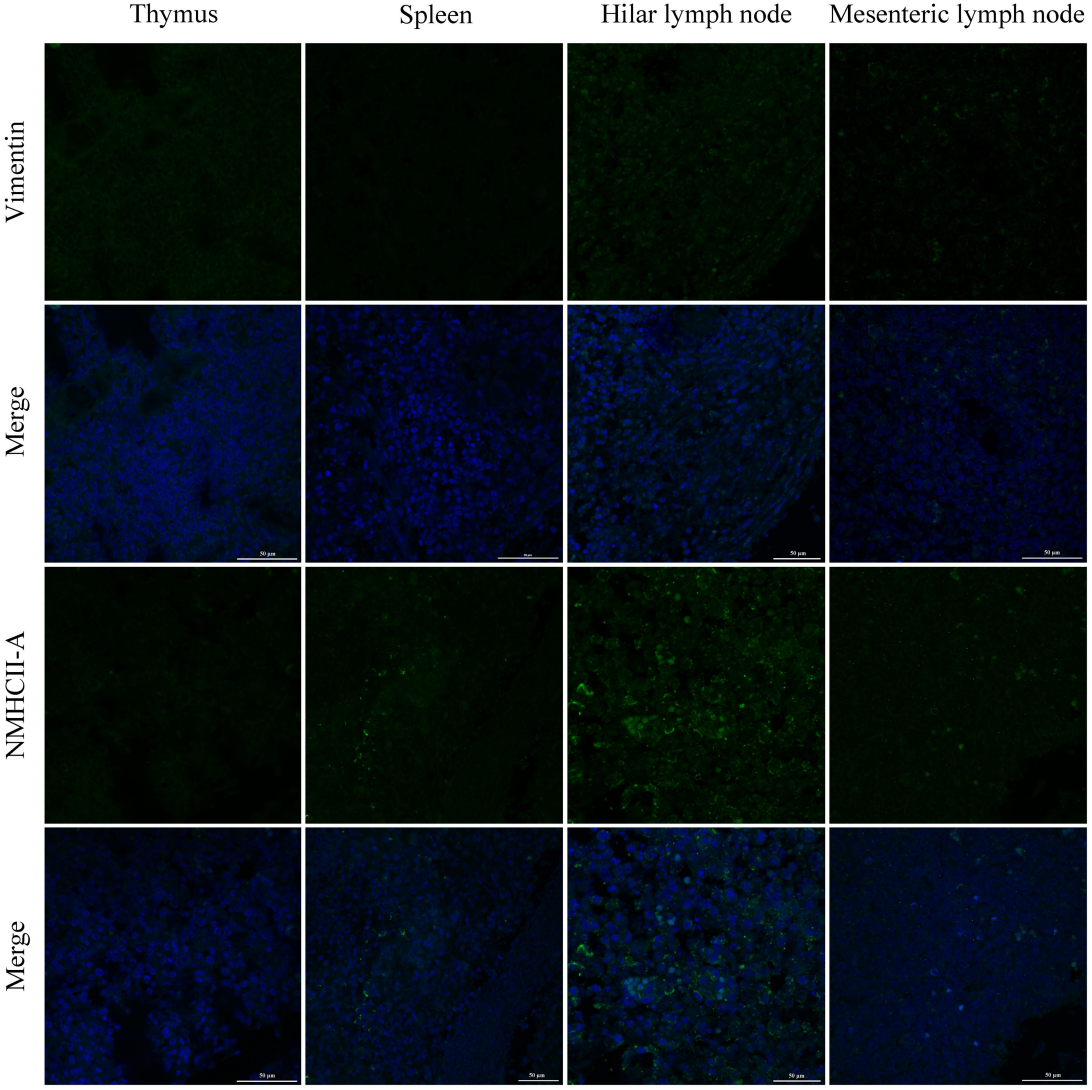
The distribution of PRRSV receptors in immune organs was analyzed by immunofluorescent staining. The receptors of Vimentin and NMHCII-A were analyzed in thymus, spleen, hilar lymph node, and mesenteric lymph node, respectively. Scale bars = 50 μm.

**Figure 7:**
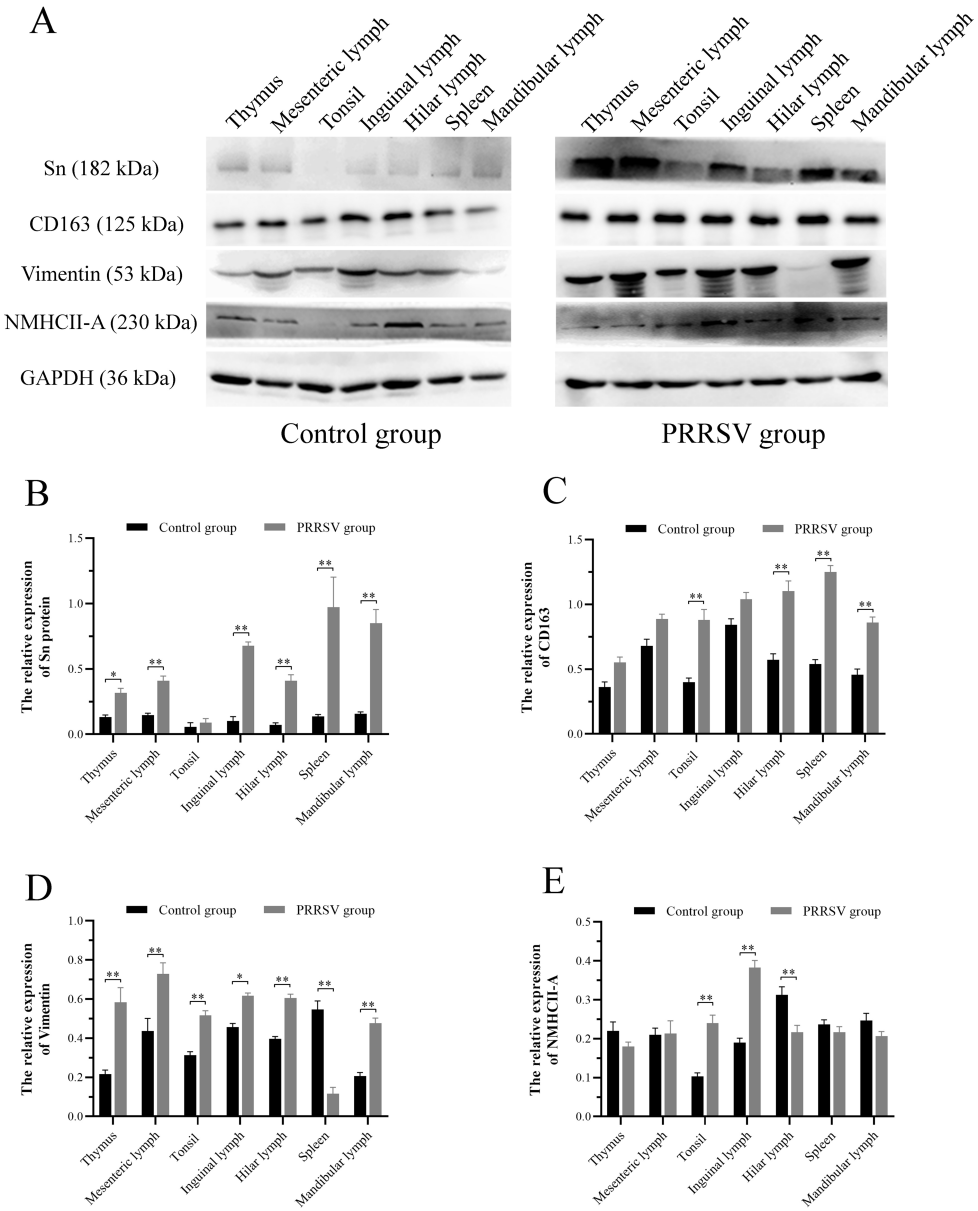
Western blot analysis was performed to measure the expression of four major receptors of PRRSV in immune organs of piglets. **(A)** The protein band of four receptors in immune organs of different groups, GAPDH protein utilized as a loading control. **(B)** The histogram represents quantification of protein levels of Sn in immune organs. **(C)** The histogram represents quantification of protein levels of CD163 in immune organs. **(D)** The histogram represents quantification of protein levels of Vimentin in immune organs. **(E)** The histogram represents quantification of protein levels of NMHCII-A in immune organs. Each value represents the mean ± SEM, *p < 0.05, **p < 0.01.

PRRSV infection of host cells involves receptor-mediated endocytosis and replication. The presence or absence of cellular receptors determines whether cells are permissive or resistant to virus infection. In this study, the distribution and expression of four important PRRSV receptors in immune organs were analyzed using IF and WB. IF results showed that CD163 was mainly distributed in the thymus, hilar lymph nodes, and mesenteric lymph nodes (Figure 5). NMHCII-A was mainly distributed in the hilar lymph nodes (Figure 6). Vimentin was expressed in all immune organs and Sn was expressed at low levels in immune tissues (Figures 5, 6). After the piglets were infected with the virus, the protein levels of the receptors were measured using WB. As shown in Figure 7, Sn expression in the thymus, mesenteric lymph nodes, inguinal lymph nodes, hilar lymph nodes, spleen, and mandibular lymph nodes was significantly increased after PRRSV infection (Figures 7A,B). CD163 expression was also increased in the tonsil, hilar lymph nodes, spleen, and mandibular lymph after PRRSV infection (Figures 7A,C). The expression of vimentin increased in all lymph nodes, but decreased in the spleen (Figures 7A,D). After PRRVS infection, NMHCII-A expression was increased in the inguinal lymph nodes and tonsil (Figures 7A,E).

## 4 Discussion

PRRS is an infectious disease that causes significant economic losses to the swine industry worldwide. Pigs are the only known natural hosts for PRRSV, and the main target of infection is fully differentiated porcine alveolar macrophages (PAMs) (22). The outcome of PRRSV infection is significantly influenced by the age of the animal, possibly due to increased innate immune resistance (14). It has been shown that piglets are more susceptible to PRRSV infection and suffer more severe and persistent infections than adults (23). However, the humoral immune response to PRRSV is activated at a low infection threshold regardless of age (14). One of the obstacles in vaccine development is the inadequate understanding of the host immune response to PRRSV (24). A previous study found that PRRSV infection persists mainly in the lungs and lymphoid tissues, leading to systemic lymphatic depletion (25). As a result, the interaction between PRRSV and the immune system can affect the host immune system (22). In this study, we found that PRRSV can cause severe pathological damage to the host immune organs, including atrophy of the thymus and general swelling of lymph nodes. Histopathological examination revealed extensive hemorrhage in the interstitial tissue and a decrease in lymphocytes count.

Infection of host cells by PRRSV is facilitated by receptor-mediated endocytosis and subsequent replication. The permissiveness or non-permissiveness of cell lines to virus infection is determined by the presence or absence of cellular receptors (16). Several cell lines, including BHK-21, PK-15, and CHO-K1, showed susceptibility to PRRSV infection upon expression of recombinant receptor proteins. Important receptors involved in virus attachment, internalization or uncoating are Sn, CD163, vimentin, and NMHCII-A (18). The macrophage-specific protein CD163 is necessary and sufficient for PRRSV infection (26). Sn is a cell adhesion molecule that has specific expression in macrophage and is predominantly localized on the surface of macrophages originating from various organs such as spleen, liver, lymph nodes, bone marrow, colon and lungs (16). Our results showed that Sn was present in immunological organs and its expression increased dramatically after PRRSV infection, suggesting that it plays a crucial role in PRRSV infection. Vanderheijden et al. suggested that Sn could serve as a potential target for immunotherapy against PRRSV infection (27). PRRSV infection correlates well with an increase in CD163 expression in cultured monocytes (28). Furthermore, CD163 expression is directly related to PRRSV infectivity in PAM cells (17). In our analysis, CD163 expression in immunological organs was associated with tissue-specific variations in virus infection. Vimentin is widely expressed in numerous cells, including monocytes, macrophages, mesenchymal cells, fibroblasts, and Sertoli cells (29, 30). Vimentin has also been shown to be localized in MARC-145 cells, which are PRRSV-susceptible cells (31). Vimentin expression has been shown to be up-regulated after PRRSV infection (32). Our results showed that vimentin expression in the spleen decreased after PRRSV infection. The location and expression of NMHCII-A, a novel PRRSV receptor, showed an inconsistent pattern after viral infection in piglets.

In summary, the primary severe lesions caused by PRRSV infection include lymph node enlargement, pulmonary hemorrhage, thymic atrophy, and hemorrhage in piglets. Histopathological examination revealed interstitial pneumonia, collapsed follicles, germinal center exhaustion, and extensive hemorrhage. Each receptor is characterized by its distribution in different immunological organs and its expression level varies after viral infection. Compared to research on the genetic variance of viruses, research into their receptors will be the goal for generating new antiviral reagents or developing more effective vaccines. However, it should be noted that our results were based on histopathological examination and morphological analyses. Further studies are required to elucidate the specific roles of host receptors in the process of PRRSV infection.

## Data Availability

The original contributions presented in the study are included in the article/Supplementary material, further inquiries can be directed to the corresponding authors.
