# Supplementary material for: Histopathological characteristics of PRRS and expression profiles of viral receptors in the piglet immune system
Source: Front Vet Sci. 2024 Nov 6;11:1428273. doi: 10.3389/fvets.2024.1428273 (PMC11576435; doi:10.3389/fvets.2024.1428273)
Supplement: Supplementary file 1 [file Data_Sheet_1.PDF]

The original figures of Western blot were provided as following:

Control group:

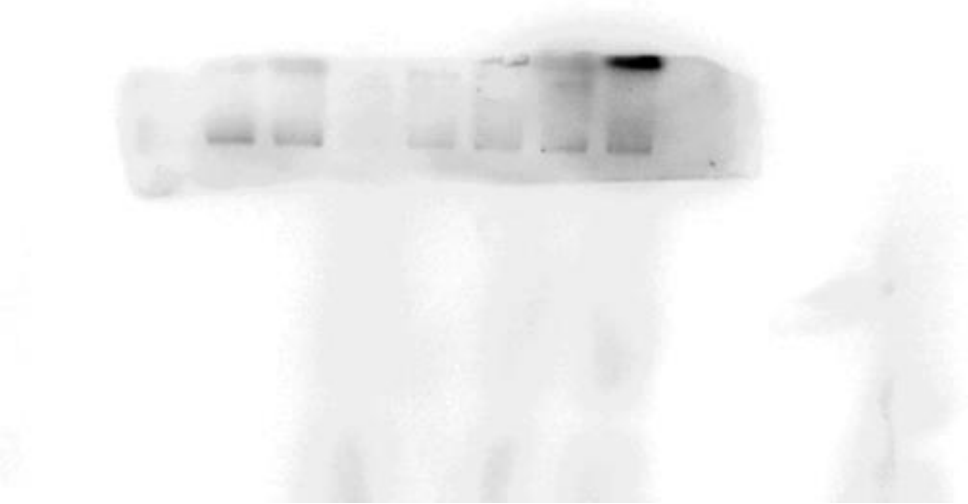

Sn

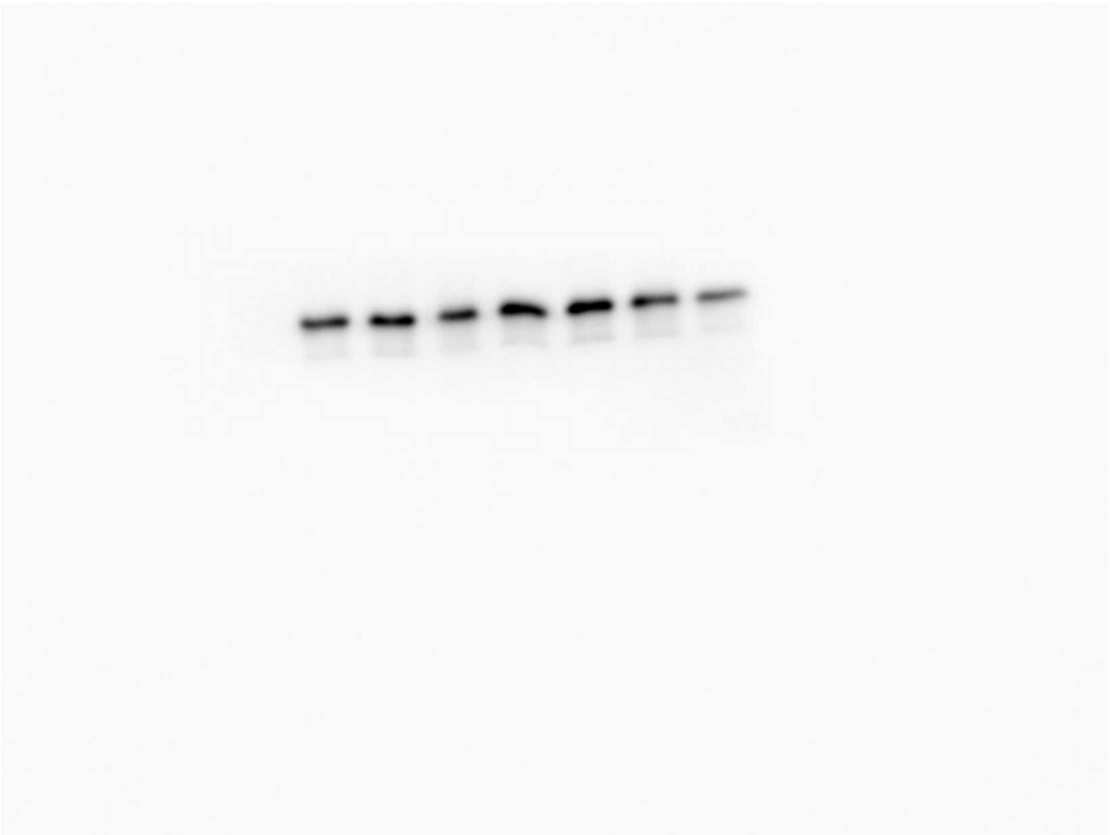

CD163

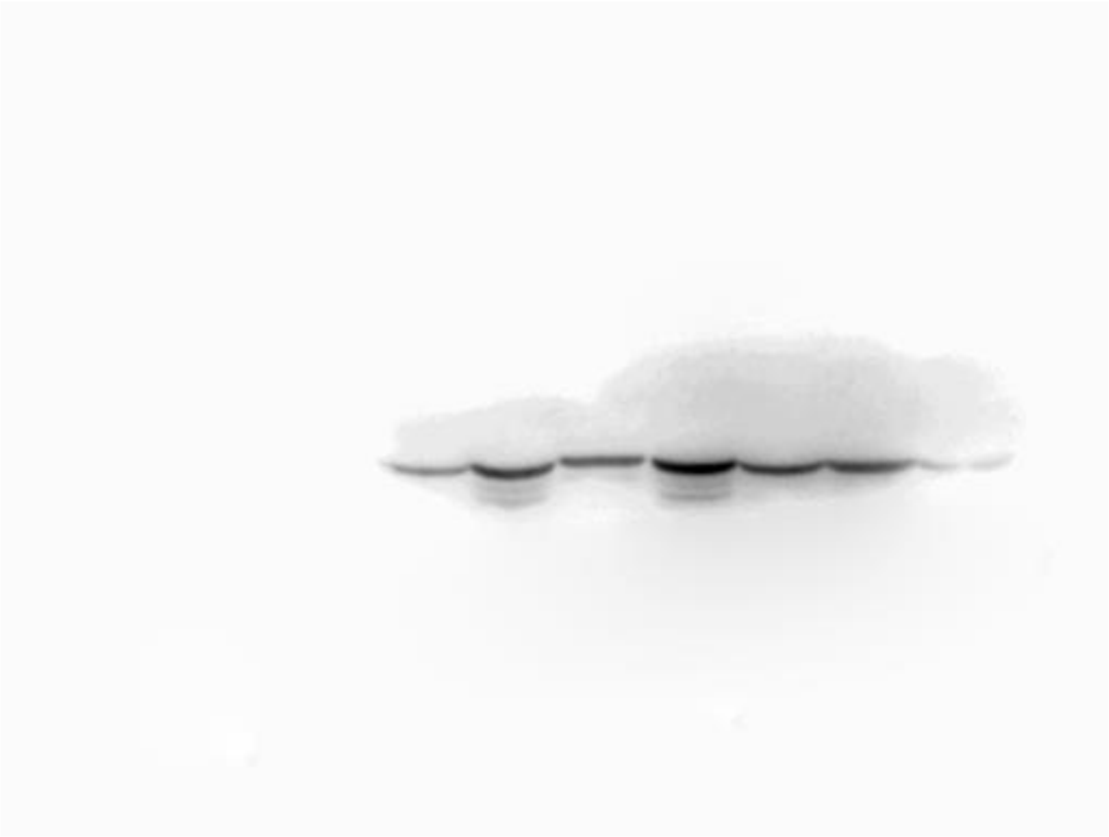

Vimentin

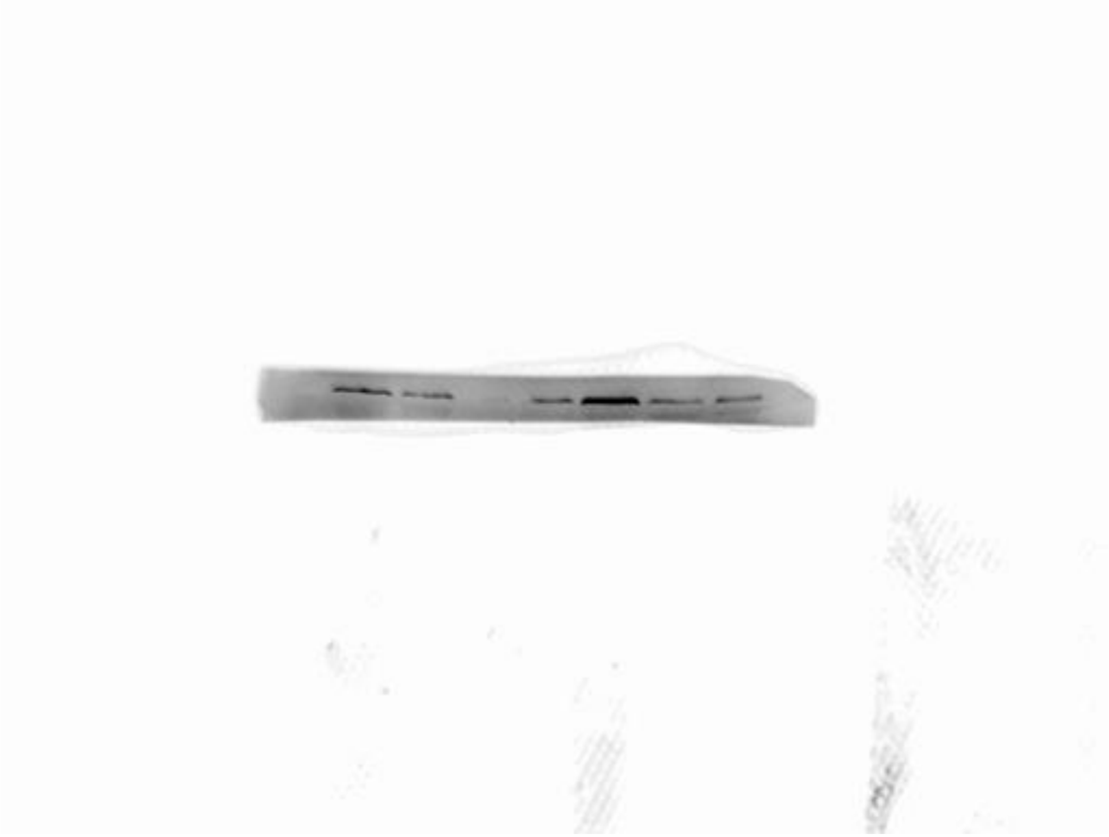

NMCHII-A

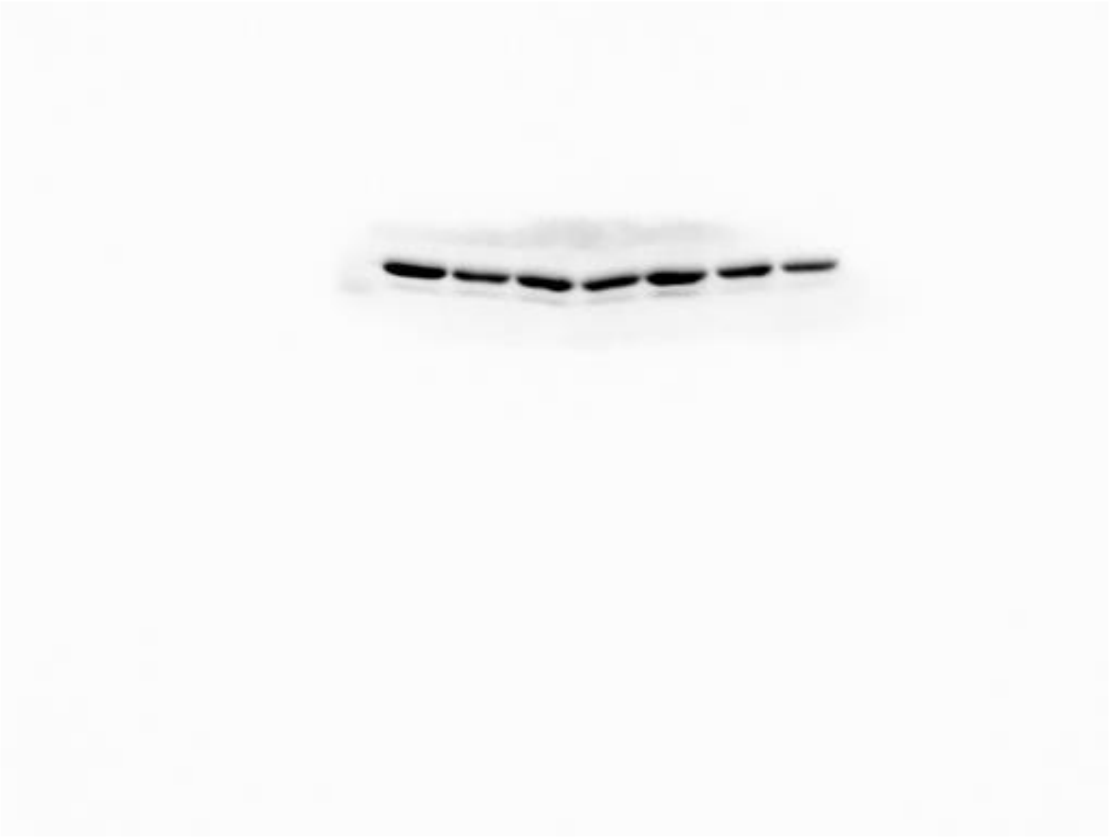

GAPDH

PRRSV Group:

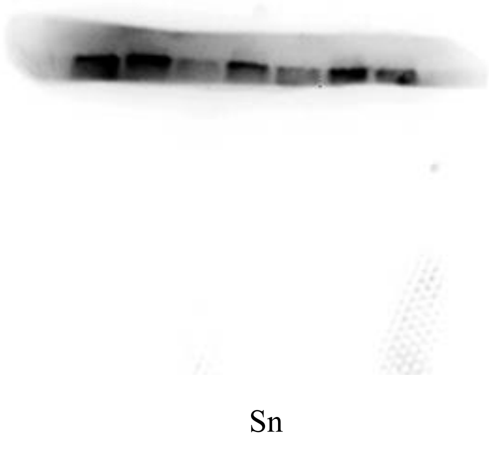

Sn

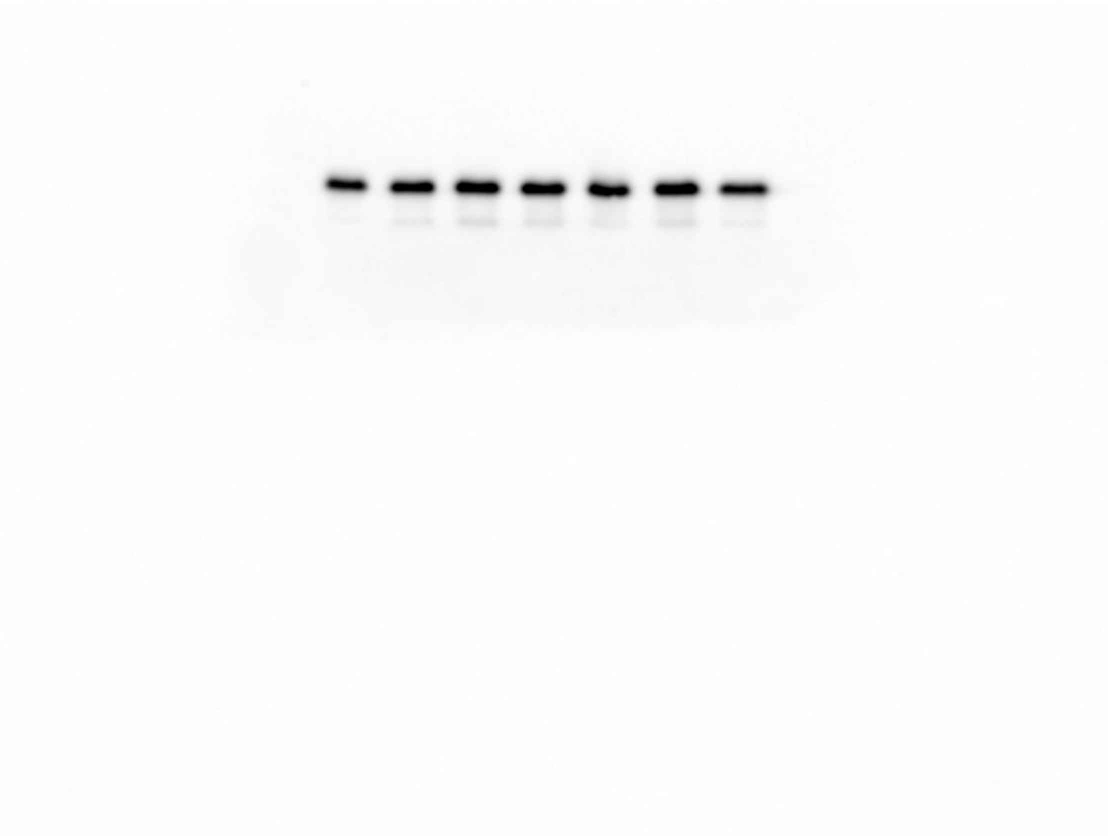

CD163

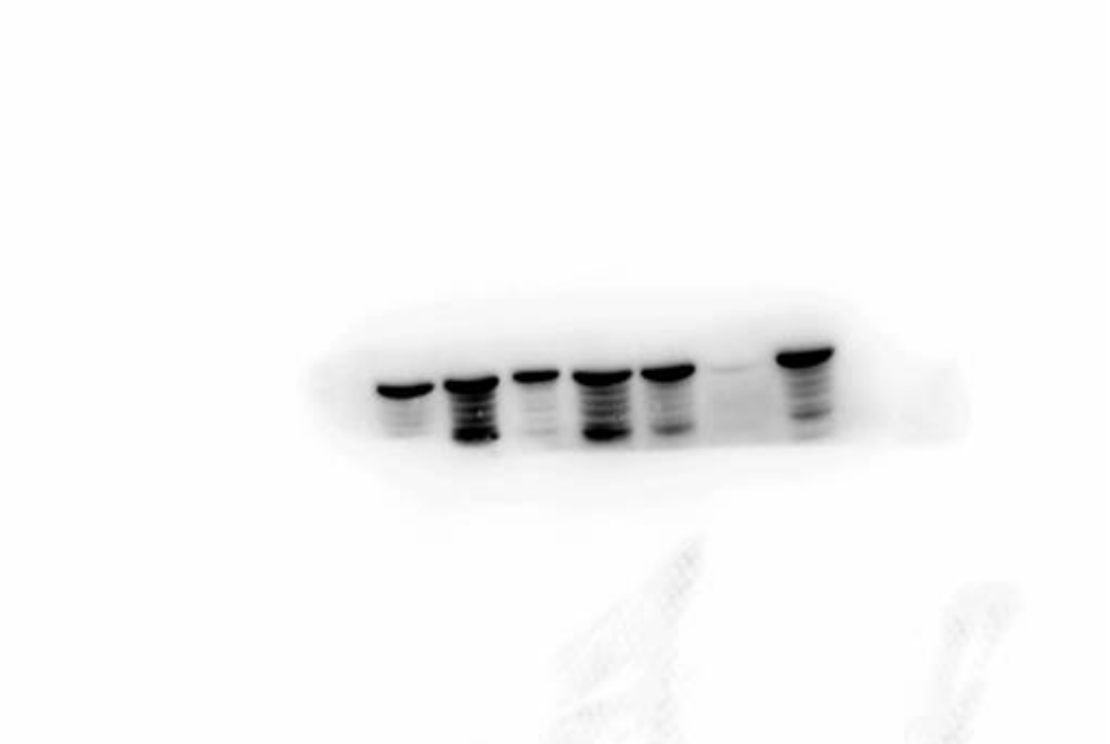

Vimentin

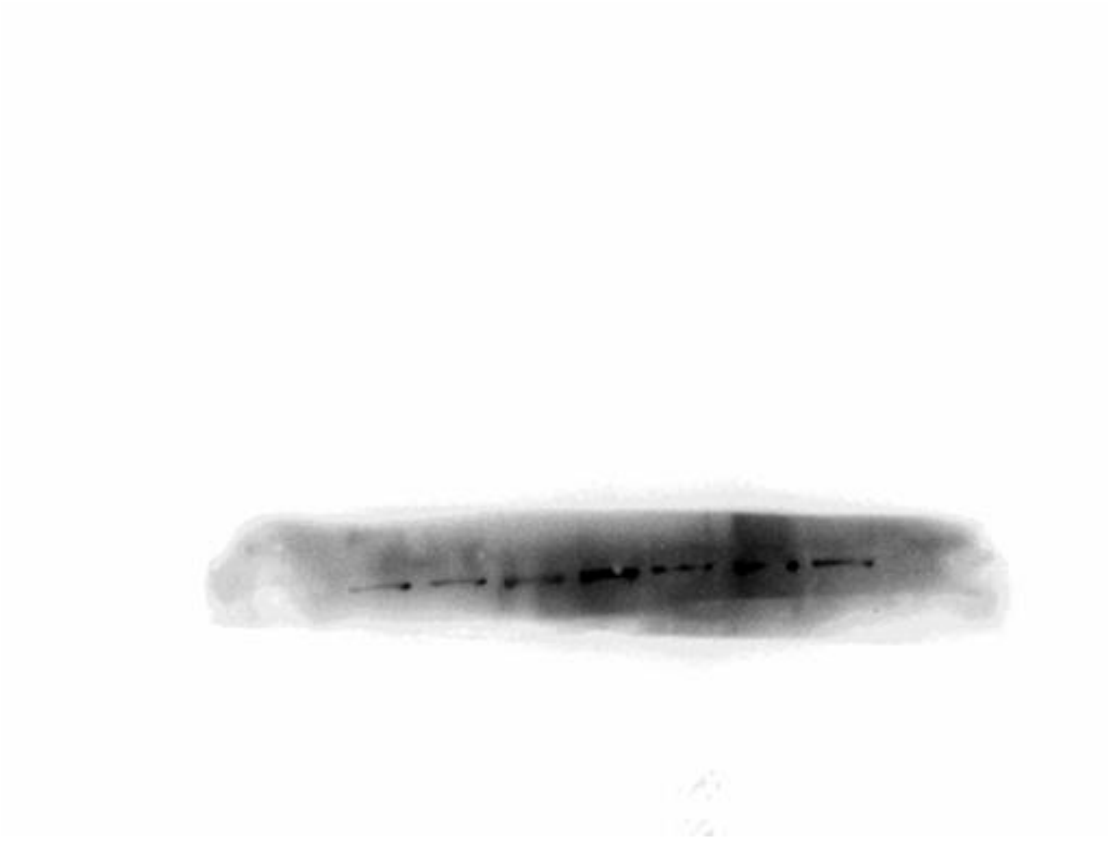

NMHII-A

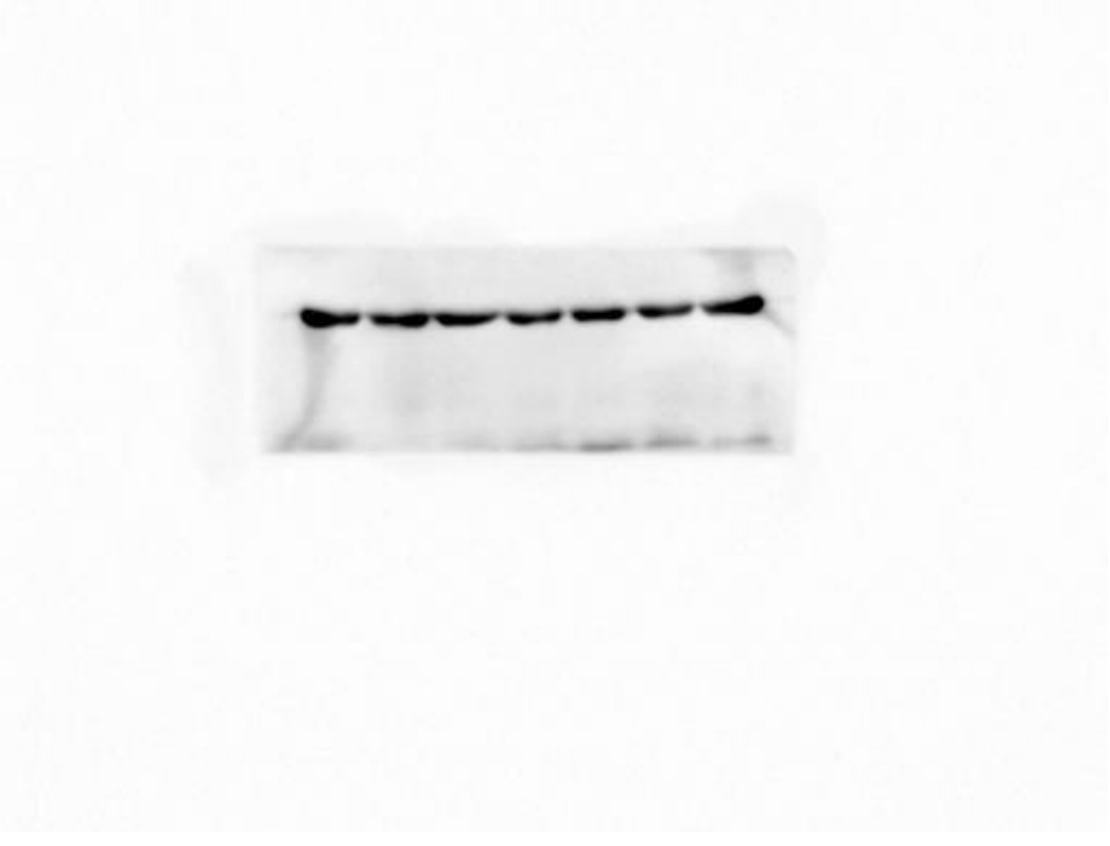

GAPDH
